# Durability test with fuel starvation using a Pt/CNF catalyst in PEMFC

**DOI:** 10.1186/1556-276X-7-34

**Published:** 2012-01-05

**Authors:** Juhae Jung, Byungil Park, Junbom Kim

**Affiliations:** 1 School of Chemical Engineering and Bioengineering, University of Ulsan, Daehak-ro 102, Nam-gu, Ulsan, 680-749, South Korea; 2 ORDEG Co. Ltd., 404 Moknae-dong, Danwon-gu, Ansan-si, Gyunggi-do, 425-100, South Korea

**Keywords:** polymer electrolyte membrane fuel cell, catalyst, carbon nanofiber, durability

## Abstract

In this study, a catalyst was synthesized on carbon nanofibers [CNFs] with a herringbone-type morphology. The Pt/CNF catalyst exhibited low hydrophilicity, low surface area, high dispersion, and high graphitic behavior on physical analysis. Electrodes (5 cm^2^) were prepared by a spray method, and the durability of the Pt/CNF was evaluated by fuel starvation. The performance was compared with a commercial catalyst before and after accelerated tests. The fuel starvation caused carbon corrosion with a reverse voltage drop. The polarization curve, EIS, and cyclic voltammetry were analyzed in order to characterize the electrochemical properties of the Pt/CNF. The performance of a membrane electrode assembly fabricated from the Pt/CNF was maintained, and the electrochemical surface area and cell resistance showed the same trend. Therefore, CNFs are expected to be a good support in polymer electrolyte membrane fuel cells.

## Introduction

Polymer electrolyte membrane fuel cells [PEMFCs] are regarded as a power source for fuel cell vehicles due to their high power density, high efficiency, and low operating temperature. The development of a fuel cell vehicle has been accelerated because of environmental problems, including global warming caused by carbon dioxide emissions and air pollution due to excessive consumption of fossil fuels. Research of the catalyst and membrane is required in order to enhance lifetime and durability for the commercialization of PEMFCs [1-4]. Currently, the corrosion of carbon is an important issue as it improves the lifetime and durability of the catalyst [5-7]. The structural breakdown by electrochemical carbon corrosion causes migration and agglomeration of the Pt particles. As a result, cell performance decreases due to a reduction in the electrochemical surface area. Carbon black [CB] is the most widely used catalyst support, but carbon corrosion occurs with long-term PEMFC operation [8,9]. Carbon corrosion is accelerated due to fuel starvation and repeated on/off cycles. When lack of fuel is generated, the electrolysis reaction of the water and carbon oxidization reaction are generated in the anode to supply the proton and electrons for the cathode oxygen reduction reaction. The carbon corrosion mechanism occurs according to the following reaction:

$$\text{C }+\;{\text{H}}_{\text{2}}\text{O }\overset{}{\underset{}{\to }}\text{ C}{\text{O}}_{\text{2}}+\text{ 4}{\text{H}}^{+}+\text{ 4}e^{-}\text{ (at 0}\text{.207 V)}.$$.

As a result, a reverse voltage is generated by alternating between the anode and cathode voltages [10-17]. Therefore, research on carbon support is required to improve durability.

Each type of carbon has a different performance and durability, but carbon characteristics can also affect the corrosion rate [2,18,19]. Cell performance will decrease due to the increase in cell resistance with reduced thickness of the catalyst layer and electric contact of the current collector caused by carbon corrosion [20]. Recently, graphitized carbon types, such as carbon nanofibers [CNFs], carbon nanotubes, and graphene, have been studied. Graphitic carbons are known to have high corrosion resistance as they have good thermal and electrochemical stability [19,21-25]. CNFs have higher electric conductivity and durability than commercial CBs as catalyst support materials [19,26]. However, it is difficult to synthesize platinum nanoparticles for loading and dispersion. CNFs with different structure and morphology have been used for electrode materials fabricated via various synthesis methods in order to achieve different surface chemistries [27-31]. CNFs are potentially suitable materials for high platinum loading and dispersion due to their many functional groups. The number of functional groups increases with increasing surface oxidation treatment time, producing a hydrophobic carbon surface which accelerates carbon corrosion [32].

In this study, we synthesized a catalyst with Pt particles of high loading and distribution on the CNFs. Membrane electrode assemblies [MEAs] were prepared using the Pt/CNF catalyst, and the performance changes caused by fuel starvation were evaluated via electrochemical analysis.

## Experimental details

### Synthesis of the Pt/CNF catalyst

The surface treatment and functionalization were carried out as follows. The CNFs (herringbone type) were placed in a flask, and H_2_SO_4_/HNO_3 _(*v*/*v *= 4:1) was added; the solution was ultrasonicated and stirred for 4 h. The CNFs were separated from the acids and washed with deionized water. A Pt salt precursor, H_2_PtCl_6_·6H_2_O (Sigma-Aldrich Corporation, St. Louis, MO, USA) was dissolved in ethylene glycol, and the CNF support was dispersed in the solution. The suspension was filtered and dried at 60°C for 4 h in a vacuum oven. The heat treatment was performed in argon atmosphere at 350°C for 2 h.

### Manufacturing of the membrane electrode assembly

In order to study the effect of the catalyst, a thin film electrode was manufactured. A mixed slurry composed of the catalyst mixed with isopropyl alcohol and a 5 wt.% Nafion ionomer solution was sprayed onto the polymer electrolyte membrane (Nafion-212 membrane). The amount of Pt was 0.4 mg/cm^2 ^on both electrodes. Table 1 lists the manufactured MEAs using a commercial catalyst and a synthesized Pt/CNF.

**Table 1:** Catalysts used for the preparation of MEAs

MEA number	Catalyst		Pt loading (mg/cm^2^)
	Anode	Cathode	
MEA-1	Pt/C	Pt/C	0.4
MEA-2	Pt/CNF	Pt/C	
MEA-3	Pt/CNF	Pt/CNF	

### Physical analysis

A thermogravimetric analyzer [TGA] (Q50, TA Instruments, New Castle, DE, USA) was used to measure the amount of Pt loaded onto the carbon support. The crystal structure and particle size of the Pt were confirmed using an X-ray diffractometer [XRD] (RAD-3C, Rigaku Corporation, Tokyo, Japan with Cu-K*α *(*λ *= 1.541 Å) at a scan rate of 1.5° min^-1^. The shape and dispersion of the Pt particles supported on the CNFs were verified by transmission electron microscopy [TEM] (JEM-2010, JEOL Ltd., Akishima, Tokyo, Japan) Brunauer-Emmett-Teller [BET] (ASAP2020, Micromeritics Instrument Co., Norcross, GA, USA) analysis was performed in order to measure the specific surface areas of the Pt/CNF and Pt/C catalysts.

### Electrochemical evaluation

The polarization curves of the unit cell were used to gauge the cell temperature at 70°C under atmospheric pressure using H_2 _and air at the anode and cathode, respectively. After obtaining the polarization curves, cyclic voltammetry [CV] was performed in the range of 0.05 to 1.2 V at a sweep rate of 50 mV/s with 20 and 100 cm^3^/min flow rates of H_2 _and N_2 _to the anode and cathode, respectively.

The durability of the assembled MEA was determined by acceleration tests using reverse potential operation under fuel starvation conditions. The acceleration experiment was operated at a current density of 400 mA/cm^2^. The hydrogen stoichiometry of the anode was maintained at 0.5. If the cell potential reached at -0.5 V, then the recovery system would be driven for 30 s under an open circuit voltage [OCV] state. In the OCV condition, the stoichiometric ratios of hydrogen and air were maintained at 1.5 and 2.0. This process was considered as 1 cycle, and experiments were repeated 200 times. After the acceleration tests, the performance curve and CV were obtained using the same method.

## Results and discussion

### Physical characteristics of the catalyst

**Figure 1:**
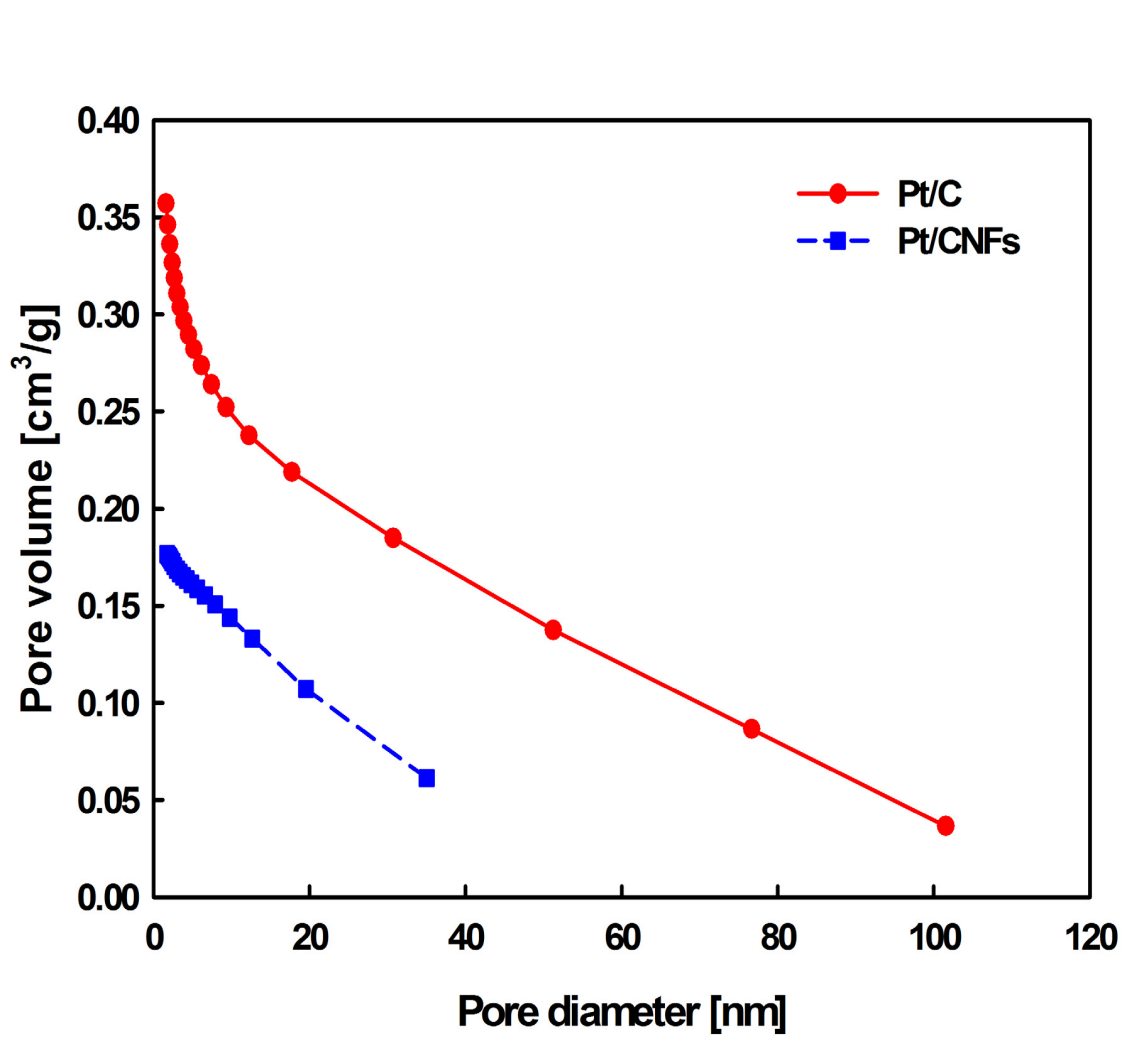
**Pore size distribution (BJH adsorption cumulative pore volume) of synthesized Pt/CNF and commercial Pt/C catalysts**.

Table 2 summarizes the physical properties of the Pt/CNF. The weight percentages of the Pt catalyst to the weight of the synthesized Pt/CNF and the commercial Pt/C were approximately 47.5 wt.% and 50.7 wt.%, respectively, based on the TGA analysis. The nitrogen adsorption and the pore size distribution were measured in the Pt/C and Pt/CNF catalysts via BET analysis. The measured BET surface areas of the Pt/CNF and Pt/C catalysts were 58.4 m^2^/g and 347.3 m^2^/g, respectively. Figure 1 illustrates the difference in pore diameter and pore volume between the Pt/CNF and Pt/C. The pore sizes of the Pt/CNF were between 1.7 and 35.0 nm, whereas those of the Pt/C catalyst were between 1.6 and 101.5 nm. In general, the pores were confirmed to be micropores (< 2 nm), mesopores (2 to 50 nm), and macropores (> 50 nm). The carbon support relieved the degradation with graphitization and low specific surface area. Pores in the catalyst layer are known to affect cell performance due to gas diffusion, mass transfer resistance, and water treatment [33,34].

**Table 2:** Properties of Pt/CNF and Pt/C catalysts

Catalyst	Weight percentage by TGA (wt.%)	Surface area by BET (m^2^/g)	Dispersion of pore size (nm)		Pt particle size (nm)	
			Minimum	Maximum	XRD	TEM
Pt/CNF	47.5	58.4	1.7	35.0	2.8	2.5
Pt/C	50.9	347.3	1.6	101.5	3.4	3.4

**Figure 2:**
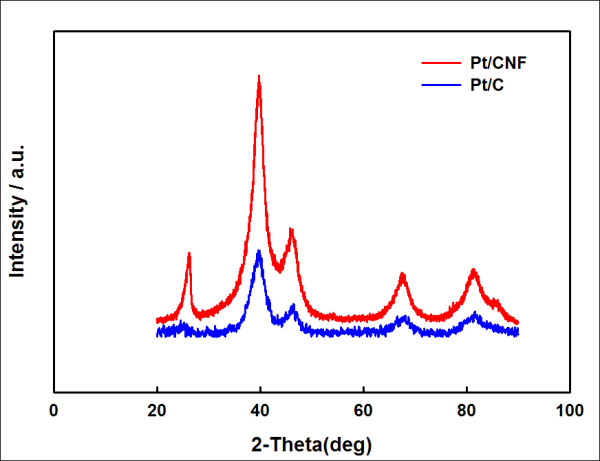
**XRD patterns of 47.5 wt.% Pt catalyst on CNFs and commercial 46.6 wt.% Pt/C catalyst**.

The morphology and crystallography of the Pt/CNF and Pt/C catalysts were studied with XRD. Figure 2 shows that face-centered cubic Pt crystal planes were observed. The average platinum particle size was calculated from the Pt (111), (200), (220), and (311) peaks using Scherrer's formula. The particle sizes in the synthesized Pt/CNF and commercial Pt/C were 2.8 and 3.4 nm, respectively. A carbon peak of (002) at 26.2° indicated that the CNF support was a graphitic structure, whereas the peak of the CB support was smaller.

**Figure 3:**
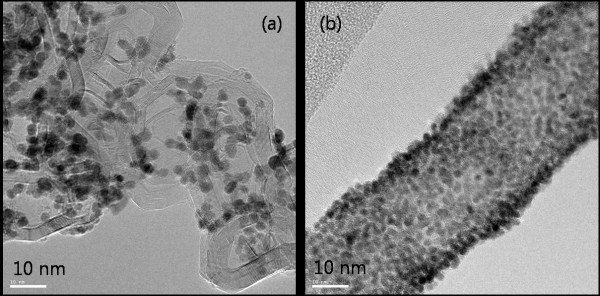
**TEM images of the (a) commercial Pt/C and (b) Pt/CNF catalysts**.

**Figure 4:**
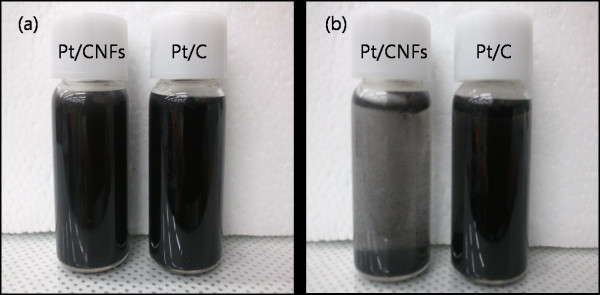
**Hydropilicity of commercial Pt/C and Pt/CNF catalysts (a) before and (b) after 2 h**.

The Pt particle size distribution and carbon type of the Pt/CNF and Pt/C catalysts were observed using TEM. Figure 3 shows that the Pt particles on the CNF supports were more distributed than those on the Pt/C catalyst. The mean particle sizes were approximately 2.5 and 3.4 nm in the Pt/CNF and Pt/C, respectively. An increase in Pt particle size can degrade cell performance with a decrease in the electrochemical surface area [ESA]. The morphology and the surface treatment with the CNF support are important due to the effect on the dispersion, particle size, and activity of Pt catalysts [31]. Figure 4 shows the hydrophilicity of the synthesized Pt/CNF and commercial Pt/C catalysts. Each catalyst was mixed with deionized water in an ultrasonic bath at room temperature. At first, the catalysts were mixed perfectly, with a change observed after 2 h. The Pt/C catalyst remained in a mixed state, while the Pt/CNF catalyst exhibited phase separation. The hydrophilicity increased with the increasing acid treatment on the carbon surface. The carbon surface was oxidized as the number of functional groups, such as carboxyls and hydroxyls, increased. However, hydrophilicity can decrease with increasing graphitization and elimination of functional groups with heat treatment. In addition, the hydrophilicity and durability of the catalyst are closely related, where the hydrophilic nature is known to degrade the durability [32].

### Electrochemical measurement with fuel starvation

**Figure 5:**
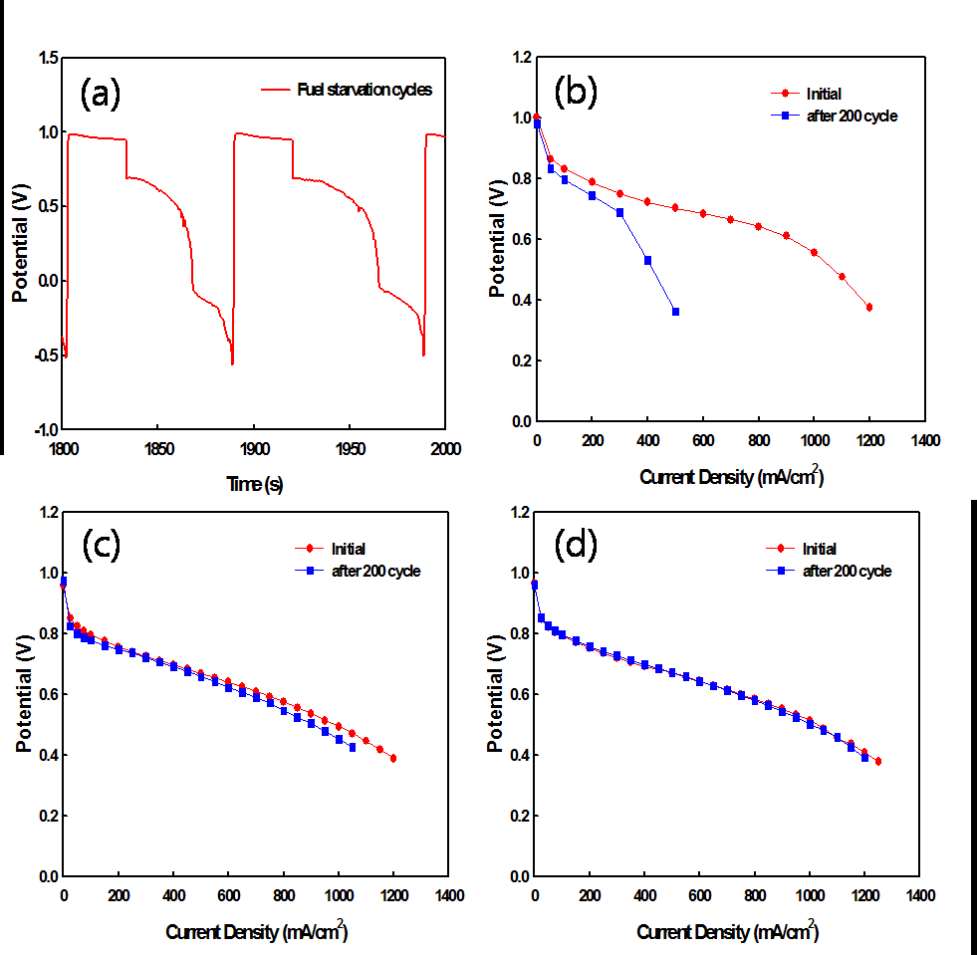
**Reverse voltage drop cycles and cell performance measurements**. (**a**) Reverse voltage drop cycles by fuel starvation and the performance of the (**b**) MEA-1, (**c**) MEA-2, and (**d**) MEA-3 before and after the fuel starvation test.

The cell performance was measured as shown in Figure 5. The change in cell voltage was determined during the fuel starvation cycles shown in Figure 5a. As shown in Figure 5b, the performance of the MEA-1 with the Pt/C catalyst rapidly decreased from 917 to 378 mA/cm^2 ^at 0.6 V after 200 cycles of fuel starvation. Activation loss and mass transfer loss were observed at a high voltage and a low voltage, respectively, as carbon corrosion was caused by applying a reverse voltage. Fuel starvation is known to cause carbon corrosion at the anode. Therefore, Figure 5c shows the performance of the MEA with Pt/CNF at only the anode. The performance of the MEA-2 was reduced by approximately 58 mA/cm^2^. Activation and mass transfer losses were observed, but they were smaller than those of MEA-1. The performance of MEA-3 with the Pt/CNF catalyst with both an anode and a cathode was only reduced by approximately 10 mA/cm^2^. Furthermore, activation loss and mass transfer loss did not occur after 200 cycles. These results indicate that MEA-3 is more durable than MEA-1 with the Pt/C catalyst because the CNF catalyst support has a strong resistance to corrosion due to its highly graphitic structure.

**Figure 6:**
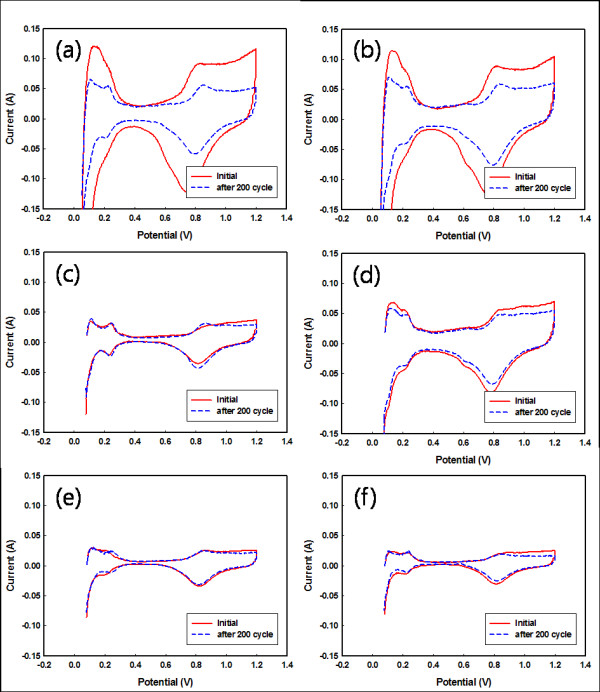
**Cyclic voltagrams of MEAs before and after the fuel starvation test**. (**a**) MEA-1 at the anode, (**b**) MEA-1 at the cathode, (**c**) MEA-2 at the anode, (**d**) MEA-2 at the cathode, (**e**) MEA-3 at the anode, and (**f**) MEA-3 at the cathode.

CV measurement was performed in order to examine changes in the ESA before and after fuel starvation. Based on the CV analysis shown in Figure 6, the ESA of the manufactured MEA-1 was initially calculated to be 59.4 and 48.5 m^2^/g at the anode (Figure 6a) and cathode (Figure 6b), respectively, but these values decreased to 33.2 and 33.3 m^2^/g after 200 cycles. Carbon corrosion caused the reduction of the ESA with the agglomeration and migration of Pt particles. Figure 6c, d shows the results of the CV on MEA-2. The ESA decreased by approximately 2.3 and 4.4 m^2^/g at the anode and cathode, respectively. These results mean that carbon corrosion resistance has occurred at both the anode and cathode when the Pt/CNF catalyst was used on the anode. As shown in Figure 6e, f, the ESA of MEA-3 was nearly unchanged in both electrodes. This tendency is similar to the cell performance results. The results of the electrochemical analysis are summarized in Table 3. Electrochemical impedance analysis measured the change in resistance at 200 mA/cm^2 ^for the manufactured MEAs. The cell resistance of MEA-1 increased from 0.103 to 0.163 Ω after 200 cycles of fuel starvation. However, MEA-2 and MEA-3 showed little change in cell resistance. Therefore, it is clear that the Pt/CNF catalyst is more durable against carbon corrosion than the Pt/C catalyst.

**Table 3:** Summary of changes before and after the fuel starvation test

MEA number	Performance at 0.6 V (mA/cm^2^)		Electrochemical surface area (m^2^/g)				Polarization resistance (Ω)	
			Anode		Cathode			
	Initial	After	Initial	After	Initial	After	Initial	After
MEA-1	917	378	59.4	33.2	48.5	33.3	0.103	0.163
MEA-2	725	667	22.1	19.8	34.2	29.8	0.096	0.104
MEA-3	746	737	17.7	17.7	14.7	14.6	0.099	0.101

## Conclusion

The 47.5 wt.% Pt/CNF catalyst was synthesized with a highly dispersed platinum. The Pt/CNF was used on the anode and on both electrodes. The MEAs were evaluated for durability against fuel starvation. After 200 cycles of reverse voltage drops, the performance of MEA-1 with Pt/C using both electrodes decreased by 59%, whereas the performance of the MEA-2 and MEA-3 was maintained. In the CV and EIS analyses, the ESA and cell resistance of the MEAs with Pt/CNF were nearly unchanged. As a result, a catalyst on a CNF support which has higher graphitization, lower specific surface area, and lower hydrophilicity has higher carbon corrosion resistance than a commercial Pt/C catalyst.

## Abbreviations

CNFs: carbon nanofibers; CV: cyclic voltage; ESA: electrochemical surface area; MEA: membrane electrode assembly; OCV: open circuit viltage; PEMFC: polymer electrolyte membrane fuel cell; TEM: transmission electron microscopy; TGA: thermogravimetric analyzer; XRD: X-ray diffractometer.

## Competing interests

The authors declare that they have no competing interests.

## Authors' contributions

JJH conceived the study, carried out all experiments, and drafted the manuscript as the first author. PBI participated in the experiment design and carried out the synthesis of catalyst and part of analysis. KJB participated in its design, coordination, and research guidance as the corresponding author. All authors read and approved the final manuscript.
